# Integrated transcriptomic analysis on chicken ovary reveals CYP21A1 affects follicle granulosa cell development and steroid hormone synthesis

**DOI:** 10.1016/j.psj.2024.103589

**Published:** 2024-02-24

**Authors:** Zhangjing You, Jingwei Yuan, Yuanmei Wang, Yanyan Sun, Aixin Ni, Yunlei Li, Hui Ma, Tenghe Ma, Jilan Chen

**Affiliations:** ⁎ State Key Laboratory of Animal Biotech Breeding, Key Laboratory of Animal (Poultry) Genetics Breeding and Reproduction of Ministry of Agriculture and Rural Affairs, Institute of Animal Sciences, Chinese Academy of Agricultural Sciences, Beijing, 100193, China; † College of Life Sciences and Food Engineering, Hebei University of Engineering, Handan, 056038, Hebei, China; ‡ Shandong Provincial Key Laboratory of Animal Biotechnology and Disease Control and Prevention, College of Animal Science and Technology, Shandong Agricultural University, Taian, 271018 China

**Keywords:** chicken, ovary transcriptome, WGCNA, granulosa cell, *CYP21A1*

## Abstract

Egg production is an economically important trait in poultry breeding and production. Follicular development was regulated by several hormones released and genes expressed in the granulosa cells, impacting the egg production and fecundity of hens. However, the molecular functions of these candidate genes that modulate these processes remain largely unknown. In the present study, bioinformatics analyses were performed to identify the candidate genes related to egg production in the ovarian tissue of White Leghorns with high egg production and Beijing You chicken with low egg production during sexual maturity and peak laying periods. The ovarian granulosa cells were used to assess the function of *CYP21A1* by transfecting with *CYP21A1*-specific small interfering RNAs (**siRNAs**) and overexpression plasmids. We identified 514 differentially expressed genes (|Log2(fold change) | >1, *P* <0.05) between the 2 chicken breeds in both laying periods. Among these genes, *CYP21A1*, which is involved in the steroid hormone biosynthesis pathway was consistently upregulated in White Leghorns. Weighted gene co-expression network analysis (**WGCNA**) further suggested that *CYP21A1* was a hub gene, which could positively respond to treatment with follicle stimulation hormone (**FSH**), affecting egg production. The interference of *CYP21A1* significantly inhibited cell proliferation and promoted cell apoptosis. Overexpression of *CYP21A1* promotes cell proliferation and inhibits cell apoptosis. Furthermore, the interference with *CYP21A1* significantly downregulated the expression of *STAR, CYP11A1, HSD3B1,* and *FSHR* and also decreased the synthesis of progesterone (**P4**) and estradiol (**E2**) in granulosa cells. Overexpression of *CYP21A1* increased the synthesis of P4 and estradiol E2 and the expression of steroid hormone synthesis-related genes in granulosa cells. Our findings provide new evidence for the biological role of *CYP21A1* on granulosa cell proliferation, apoptosis, and steroid hormone synthesis, which lays the theoretical basis for improving egg production.

## INTRODUCTION

Egg production is one of the most important traits in the poultry industry. Follicular development is a key determinant of the egg production in poultry. Efficient egg laying mainly depends on the active prehierarchical follicle recruitment, follicular selection and differentiation, preovulatory follicular hierarchy, and ovulation, which are complex biological processes regulated by endocrine hormones synthesized in the hypothalamus-pituitary-gonad axis and various paracrine or autocrine factors in the follicles (Shen et al., 2021). An intact follicle consists of 1 oocyte and 2 main types of somatic cells, theca cells and granulosa cells (**GCs**) (Yoshimura and Koga, 1982; Wulff et al., 2001). The proliferation of granulosa cells promotes follicular maturation, whereas the apoptosis of granulosa cells triggers follicular atresia. The granulosa cells are responsible for secretion of steroid hormones, such as progesterone (**P4**), providing a critical microenvironment for follicle growth and maintaining endocrine system homeostasis (Zhang et al., 2019b; An et al., 2020), and then induce the mature oocyte production, and ovulation. Therefore, understanding the mechanisms of functional genes related to follicular development in the granulosa cells is critical for improving egg production traits.

Exploring the molecular mechanisms of follicle development has always been a hot research topic among the various reproductive traits. Recently, high-throughput sequencing and other molecular biological technologies have provided new ideas and approaches for poultry research. By analyzing the transcriptome data of ovarian tissues from 3 hens with high rates of egg laying and 3 hens with low rates, *STC1* was found to play an important role in chicken follicle development (Sun et al., 2023). Similarly, *COL4A2* is a key candidate gene that contributed to the chicken follicular development (Sun et al., 2022). During the developmental process of ovarian pre-hierarchical follicle, *RAC1* and *SLC5A5* exert a positive effect on the granulosa cells proliferation, differentiation, and steroidogenesis of the follicles (Tyasi et al., 2020; Shen et al., 2024). In addition, previous studies have found that *BMP15* and its SNPs are associated with the development of ovarian follicles, especially in the development of primordial follicles (Han et al., 2015). White Leghorn and Beijing You chicken are 2 distinct chicken breeds, in which White Leghorn is a high-yielding laying chicken that has undergone intensive genetic selection, while Beijing You chicken is a Chinese indigenous breed with superior meat and egg quality, but the egg production is significantly lower than that of White Leghorn (Silversides, 2010; Liu et al., 2021; Yuan et al., 2023). The 2 breeds could be the typical chicken breed in the study of mechanism related to the egg production. Subsequently, the ovaries of 2 breeds were used to search for genes associated with egg production.

Cytochrome P450 monooxygenase, a type of heme-thiolate protein, belongs to a multigene superfamily with an ancient origin. Its carbon-monoxide-binding form has a signature absorption peak at 450 nm and is referred to as P450 (Werck-Reichhart and Feyereisen, 2000). As one of the largest enzyme protein superfamilies, P450 family genes have been discovered in a variety of organisms including animals, plants, fungi, protists, archaea and even viruses (Nelson, 2009). Inside the cell, cytochrome P450 enzymes are found on the slippery endoplasmic reticulum that synthesize proteins and on the mitochondria that transfer energy, respectively. Several members of this subfamily have been identified, such as *CYP11A1, CYP11B1, CYP17A1, CYP19A1*, and *CYP21A1*. (Zhang et al., 2012). Members of this subfamily are involved in the regulation of many key physiological processes in mammals, including energy metabolism, immune regulation, stress adaptation, homeostasis, sexual differentiation, and gonadal formation and maturation of sexual organs (Guo et al., 2003; Shih et al., 2011). *CYP11A1* is an imperative marker in the steroid synthesis pathway (Li and Johnson, 1993). As the first and rate-limiting step of steroid biosynthesis, *CYP11A1* catalyzes the conversion of cholesterol to pregnenolone, which is transformed into various forms of mineralocorticoids, glucocorticoids and androgens in adrenal cortices, progestins and estrogens in ovaries and placentae (Guo et al., 2003). *CYP17A1* and *CYP19A1* encode aromatase, which is a rate-limiting enzyme of estradiol (**E2**) synthesis. They are essential for converting androgens into estrogens in granulosa cells regulating the follicular development (Richards, 1980). *CYP21A1* was reported to be in the granulosa layer of ovulatory follicles and fetal testis and promoted the ovulatory process in mice (Hu et al., 2007; Espey et al., 2008). Extra-adrenal induction of *CYP21A1* could ameliorate steroid metabolism (Naiki et al., 2016). In chicken, *CYP21A1* was located on chromosome 16. Previous study identified a putative region under selection containing *CYP21A1* that related to the immune traits since almost all the genes that are currently mapped to chicken chromosome 16 play a prominent role in immune response (Rostamzadeh-Mahdabi et al., 2021). Nevertheless, little is known about whether *CYP21A1* exerts an effect on follicular development in avian species.

In this study, the transcriptome analysis of ovary tissues of White Leghorn and Beijing You chicken during sexual maturity and peak laying periods was implemented to reveal the important genes related to follicular development, and then the candidate gene *CYP21A1* was selected to explore its functions in chicken ovarian granulosa cells. The findings would contribute to a better understanding of the molecular mechanisms underlying follicular development.

## MATERIALS AND METHODS

### Ethics Statement

All experimental procedures involving the use of animals were conducted according to the Guidelines for Experiment established by the Science Research Department of the Institute of Animal Science, Chinese Academy of Agriculture Sciences (**IAS-CAAS**), and ethical approval was obtained from the Animal Ethics Committee of the IAS-CAAS (No. IAS 2022-35).

### Management of Experimental Birds and Sample Collections

In current study, White Leghorns and Beijing You chicken from the same batch raised in the same environmental conditions were selected as experimental objects. Birds were kept in individual cages, and egg numbers were daily collected. When the laying rate of the population reached 5% (7.33% at 22 wks of age), 6 birds each from 2 breeds were selected for sampling based on the average of pubic space, which was measured within 60 birds each randomly selected from 2 breeds. Peak laying periods (28∼40 wks of age) were characterized based on the laying rate > 80%. At 35 wks of age, 6 individuals each from 2 breeds were selected for sampling based on the average egg numbers produced by each breed. For the tissue harvesting, the ovary was isolated from the abdominal cavity. All visible follicles including white cortical follicles that are 1 to 5 mm in diameter, small yellow follicles of 5 to 8 mm in diameter were carefully excluded as previously reported (Onagbesan et al., 2009). The harvested tissues were immediately frozen in liquid nitrogen for RNA sequencing.

### RNA Isolation and RNA-seq Library Preparation

Total RNA was extracted using TRIzol Reagent (Invitrogen, Carlsbad, CA) following the manufacturer's instructions. The RNA integrity and quality were determined by agarose gel electrophoresis and Nanodrop 2000 nucleic acid and protein detector (Thermo Fisher, MA). Three micrograms of total RNA were used for the construction of mRNA library, and then the ribosomal RNA was removed from total RNA with TruSeq Stranded Total RNA Library Prep Gold Kits (Illumina, San Diego). RNA sequencing libraries were constructed for paired-end sequencing according to the instructions of NEB Next Ultra Directional RNA Library Prep Kit for Illumina (NEB, Ipswich, MA). Sequencing was performed using Novaseq 6000 (Illumina) to generate 150bp paired-end reads.

### Differential Expression Analyses and Functional Annotations

The adapter contamination, low-quality reads (Phred Quality Score < 5%), reads with poly-N > 5%, and reads matched to rRNA were filtered out using in-house Perl scripts to generate clean reads, which were aligned to the chicken reference genome Gallus gallus 6.0 using Hisat2 (v2.1.0) (Pertea et al., 2016) with default parameters, and then the mapped reads were assembled by StringTie (v2.1.5) (Pertea et al., 2016) with gene transfer format file of Ensemble gene annotation. Gene count matrix was generated using the featureCounts software (v2.0.3) (Wu et al., 2006), and the resulting count data was fed in DESeq2 (v1.16.1) to detect differentially expressed genes (**DEGs**) (Love, et al., 2014). |Log2(fold change) | >1 and P <0.05 were the criteria used to identify the DEGs in the corresponding comparison. Gene ontology (**GO**) and Kyoto Encyclopedia of Genes and Genomes (**KEGG**) analyses were performed in KOBAS (Bu et al., 2021), and *P* < 0.05 was considered as the significant threshold.

### Weighted Gene Co-expression Network Analysis (**WGCNA**)

Weighted Gene Co-expression Network Analysis (WGCNA) utilizes a systematic approach to cluster genes that have similar expression patterns, transforming the expression of genes into modules (Langfelder and Horvath, 2008). WGCNA package in R software was used to construct and visualize the network using genes with the top 75% median absolute deviation (**MAD**), which expressed as $n^{-1}\sum_{i=1}^{n}\left|O_{i}-\bar{O}\right|$, n is the number of observations, O is the “pairwise-matched observations that are judged to be reliable”, the $\bar{O}$ is “true” mean of the observations. The MAD was calculated in R software. First, an appropriate “soft-thresholding” value was selected by plotting the strength of the correlation against a series of soft threshold powers (from 1 to 30), with a signed pairwise correlation matrix generated by Pearson's product moment correlation. The correlation matrix was subsequently transformed into an adjacency matrix, in which node and edge corresponded to genes and the connection strength between genes, respectively. Each adjacency matrix was normalized using a topological overlap function. Hierarchical clustering was performed using average linkage. The hierarchical cluster tree was cut into modules using the dynamic tree cut algorithm with a minimum module size of 50 genes. We merged modules if the correlation between their eigengenes (defined as the first principal component of their gene expression values) was greater or equal to 0.25. After obtaining modules, the module eigengene that summarized as the first principal component of expression data was calculated with the “Module Eigengenes” function. Correlation analysis between a module and the breed was performed with the “corPvalueStudent” function based on the module eigengene, and *P* < 0.01 was set for statistical significance. The gene significance (**GS**) and module membership (**MM**) values were also generated to screen hub genes when GS > 0.5 and MM > 0.5.

### Cell Culture

The granulosa cells were isolated and cultured by Gilbert's established method (Gilbert et al., 1977). Briefly, the hierarchical follicles (6∼10mm in diameter) were obtained from White Leghorn hens, and then they were placed in phosphate-buffered saline (**PBS**) containing 3% double antibiotics (Solarbio, Beijing, China) to remove the residual connective tissue and attached blood filaments. The outer layer of the follicle membrane was peeled off with pointed tweezers. All granulosa cells were assembled in 1.5 mL centrifuge tubes, ground into a homogeneous paste by small tissue scissors, mixed in 15 mL centrifuge tubes, and digested in a 37°C environment with 5 mL 0.25% trypsin (Solarbio) for 8 min. After the digestion process, single cells were obtained by filtration through 200 μm sieve. After centrifugation twice, the suspended cells in Medium 199 (Gibco, Waltham, MA) complete culture medium containing 5% fetal bovine serum (Gibco) and 1% double antibiotics were spread in a 6-well plate. The number of cells was detected using trypan blue. Cells were cultured at 37.5°C in an atmosphere of water-saturated 5% CO_2._

### Identification of Ovarian Granulosa Cells

The trypsin-digested granulosa cells were inoculated on the cell crawlers and incubated for 24 h. The cell crawlers were gently washed with PBS for 3 min and fixed with 4% paraformaldehyde at 4°C for 30 min. This was followed by incubation of permeabilization solution (0.2% Tritonx-100) on a shaker at room temperature for 15 min, and then washed with PBS for 3 min. The cell crawlers were blocked with 3% normal goat serum (Bbi Biotech, Shanghai, China) for 30 min, then 100 μL FSHR rabbit polyclonal antibody (1:50) was added to incubate the cells at 37°C for 2 h, and then the cells were washed with PBS for 5 min. After that, 100 μL FITC-labeled mouse antirabbit antibody (1:100) was added and incubated at 37°C for 1h. The slices were sealed with an antifluorescent attenuating blocker (DAPI) and photographed by laser confocal.

### Follicle Stimulation Hormone Treatment

The cells were cultured with serum-free M199 medium in the absence and presence of different concentrations: 5, 50 and 80 ng/mL of FSH (Sigma-Aldrich, St. Louis, MO). After 24 h, total RNA from granulosa cells was extracted from each group.

### Plasmid Construction, siRNA and Cell Transfection

The coding sequence of *CYP21A1* was amplified from chicken follicle cDNA by PCR using gene-specific clone primers. The PCR product was cloned into the pcDNA3.1-EGFP vector (Invitrogen) within NheI and EcoRI sites. The overexpression vector of *CYP21A1* was confirmed by agarose gel electrophoresis and DNA sequencing. The siRNA specifically against chicken *CYP21A1* was obtained from GenePharma (Shanghai, China), and a nonspecific duplex was used as the control. The siRNAs used in this study as follows:5′-GCACAUGGCCCUCGUGGAUUUTT-3′. Granulosa cells were planted on 6-well plates for the transient transfection experiments. The cells were transfected with the pcDNA3.1-*CYP21A1* overexpression plasmid and siRNA-*CYP21A1* (2500 ng/well) using Lipofection LTX and Plus Reagent, respectively. Forty-eight hours after transfection, the cells were lysed for RNA extraction.

### RNA Extraction, cDNA Synthesis and Real-time Quantitative Polymerase Chain Reaction

Total RNA was extracted from granulosa cells using TRIzol reagent according to the manufacturer^’^s guidelines. The purity and concentration of the RNA were determined by measuring the ratio between the absorbance at 260 nm and 280 nm by a spectrophotometer (Thermo Fisher). The cDNA was synthesized by reverse transcription using HiScripIII First Strand cDNA Synthesis Kit (Novizan, Nanjing, China) containing gDNA wiper. Real-time quantitative PCR (**qRT-PCR**) was conducted on an MX3000p instrument using the SYBR premix ExTAq. The reaction protocol was: initial denaturation at 95°C for 2 min, denaturation at 95°C for 3 s, primer annealing at 60°C for 32 s, primer extension at 95°C for 15 s and final extension 60°C for 1 min in 40 cycles of amplification. Primer sequences are listed in Table 1. Beta-actin was applied as an internal parameter, and each sample was repeated 3 times. The relative expression value was calculated by 2^−△△Ct^ method.

**Table 1:** Primers used for qRT-PCR.

Genes	Accession number (GenBank)	Product size (bp)	Strand	Sequence (5′-3′)	Annealing temperature(°C)
*β-actin*	NM_205518	150	F	TGCTGTGTTCCCATCTATCG	60
			R	TTGGTGACAATACCGTGTTCA	
*CYP21A1*	NM_001099358.2	145	F	ATGAGTTCCTGCCCGAGCG	60
			R	CCACACCTCCAGCAGTTCCACCACA	
*STAR*	NM_204686.3	148	F	AATCGCTGTAGGATGTGCCC	60
			R	TGGTTGATGATGGTCTTTGGCAGC	
*FSHR*	NM_205079.2	158	F	ACCAATGCCACAGAACTGAGA	60
			R	TGTAGTTTGGGAAGGCTGGA	
*HSD3B1*	XM_015294362.4	104	F	GCCAAAGAGGAGCAAACCAGAG	60
			R	TCCAGCAGTAAGCGAACGATCC	
*CYP11A1*	NM_001001756.2	158	F	TCCGCCACCTCAACACCAAGA	60
			R	CACAAGGAGGCTGAAGAGGATGC	

### Cell Counting Kit-8 Assay

The granulosa cells were cultured in a 96-well plate and in a growth medium. A cell counting kit-8 (Beyotime, Shanghai, China) was used for this experiment. Cell proliferation was monitored at 0, 24, 48 and 72 h after transfection. Each well was incubated with 10 μL cell counting kit-8 (**CCK-8**) reagent. After 2h of incubation, the optical density values were obtained using a microplate reader (Thermo Fisher) at 450 nm.

### Flow Cytometric Analysis

The rate of granulosa cells’ apoptosis was detected by flow cytometry analysis. The granulosa cells were cultured in 12-well plates. Then the cells were washed with PBS and lysed with 0.25% trypsin for 2min and were collected 48 h after transfection. Then the cell suspension was centrifuged at 300 x g for 5 min, and the supernatant was discarded and the remaining cells were resuspended with 100 μL 1 × binding buffer. Subsequentially, 5 μL of Annexin V-FITC (BD Biosciences, Franklin Lakes, NJ) and 5 μL propidium iodide (BD Biosciences) were added for 5 min in the dark condition. Furthermore, 400 μL 1 × binding buffer was added to each sample and evenly mixed with a vortex mixer before the analysis. Then the flow cytometer was used for analysis, and the data were processed using FlowJo10.8.1 software (BD Biosciences).

### Enzyme-linked Immunosorbent Assay for Steroid Hormones

The granulosa cells were transfected in 12-well plates for 48 h, and then cell supernatants were collected. Concentrations of P4 and estradiol E2 were determined using the Chicken P4 and E2 Enzyme-linked immunosorbent assay (**ELISA**) kit (Meimian, Jiangsu, China), respectively, according to the manufacturer's instructions. The sensitivity of ELISA kits of P4, and E2 were typically less than 10 pmol/L and 1.0 pg/mL tolerance within batch and tolerance between batches of CV < 10% and no cross-reactivity for all ELISA kits.

### Statistical Analysis

All data are presented as the mean ± standard error. Statistical analysis was performed using SPSS 19.0 (IBM Corp, Armonk, NY). We compared data between 2 groups with students’ t-test and those among more than 2 groups with a one-way ANOVA. Each experiment was repeated 3 times. *P* < 0.05 was considered as threshold of significantly difference.

## RESULTS

### Identification and Functional Annotation of Differentially Expressed Genes

**Figure 1:**
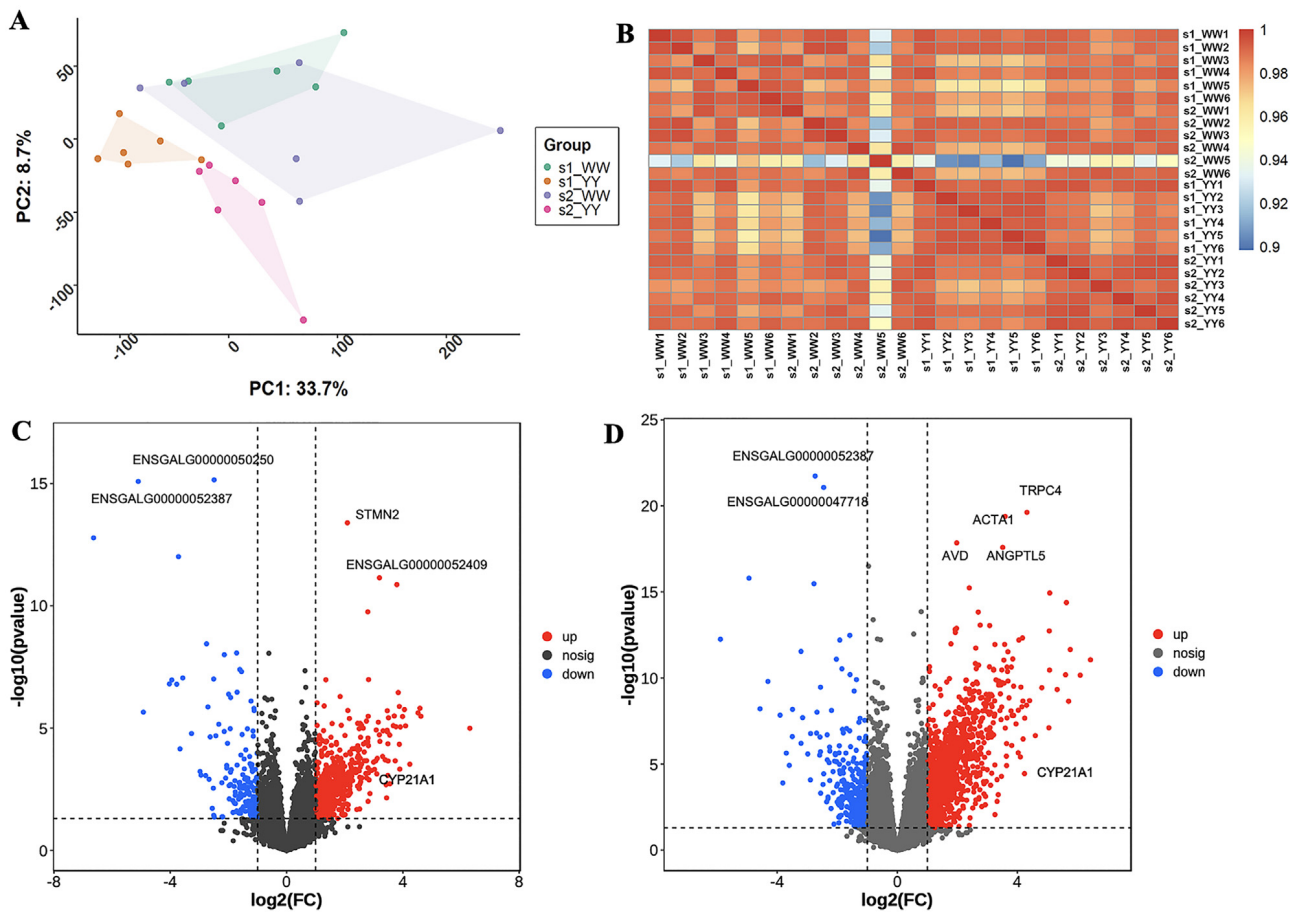
Transcriptomic analysis for ovary of White Leghorn and Beijing You chicken in sexual maturity and peak laying periods. (A) PCA plot for ovary transcriptomes of 2 breeds in 2 laying periods. (B) The global gene expression correlation of different samples. (C) Volcano map of differentially expressed genes between White Leghorns and Beijing You Chicken at sexual maturity. (D) Volcano map of differentially expressed genes between White Leghorn and Beijing You chicken in peak laying periods. WW and YY denote White Leghorn and Beijing You chicken, respectively; s1 and s2 denote sexual maturity and peak laying periods, respectively.

For the phenotypic value of chicken population used in present study, the age at first egg of White Leghorn (156.49±7.30) was 21.35 d earlier than Beijing You chicken (177.84±9.85). At 35 wk of age, White Leghorn (78.68±13.14) laid 27.77 eggs more than Beijing you chicken (50.91±12.23). Prior to the analysis of differentially expressed genes, principal component analyses were performed to compare the expression of expressed genes between 2 breeds in the 2 laying stages, respectively. We found that the global gene expression of the 2 breeds was clearly separated. The gene expression of Beijing You chicken can be separated between sexual maturity and peak laying periods, while interaction occurred in White Leghorns (Figure 1A). By analyzing the correlation of global gene expression among samples, we found that the correlations were strong, as high as more than 0.9 (Figure 1B). These results indicated that the expression of ovarian genes in different breeds and different laying periods was highly conservative, and the specific expression of variant genes might be the genetic basis for the differences in egg production traits between 2 chicken breeds. By analyzing RNA-seq data, we found a total of 2,499 DEGs between the 2 breeds (*P* < 0.05, log2|FC|≥1) in the sexual maturity period. Among these DEGs, 1,387 genes were upregulated, and 399 genes were downregulated (Figure 1C). At the peak of laying period, a total of 713 DEGs were identified between the 2 breeds (*P* < 0.05, log2|FC|≥1), of which 534 genes were upregulated and 179 genes were downregulated (Figure 1D). After overlapping the DEGs in the 2 laying stages, we identified 514 common DEGs, including 410 upregulated genes and 104 downregulated genes (Supplementary Table S1). KEGG enrichment analysis revealed that the commonly upregulated DEGs were mainly enriched in ECM-receptor interaction pathway, focal adhesion pathway and vascular smooth muscle contraction pathway (Supplementary Table S2). The commonly upregulated genes were also annotated to various GO terms, such as extracellular matrix organization, cell adhesion, collagen fibril organization, and endodermal cell differentiation multicellular organism process in biological processes (**BPs**) and cellular components (**CCs**) of extracellular space, basement membrane and focal adhesion and molecular functions (**MFs**) of calcium ion binding, extracellular matrix structural constituent, protein binging, collagen binding and structural constituent of muscle (Supplementary Table S3). Notably, the steroid hormone biosynthesis related to ovarian follicle development was also enriched by DEGs, including *CYP21A1* and *HSD11B2,* suggested that *CYP21A1* might be a functional gene affecting egg production traits.

### Weighted Gene Co-expression Network Analysis (WGCNA)

**Figure 2:**
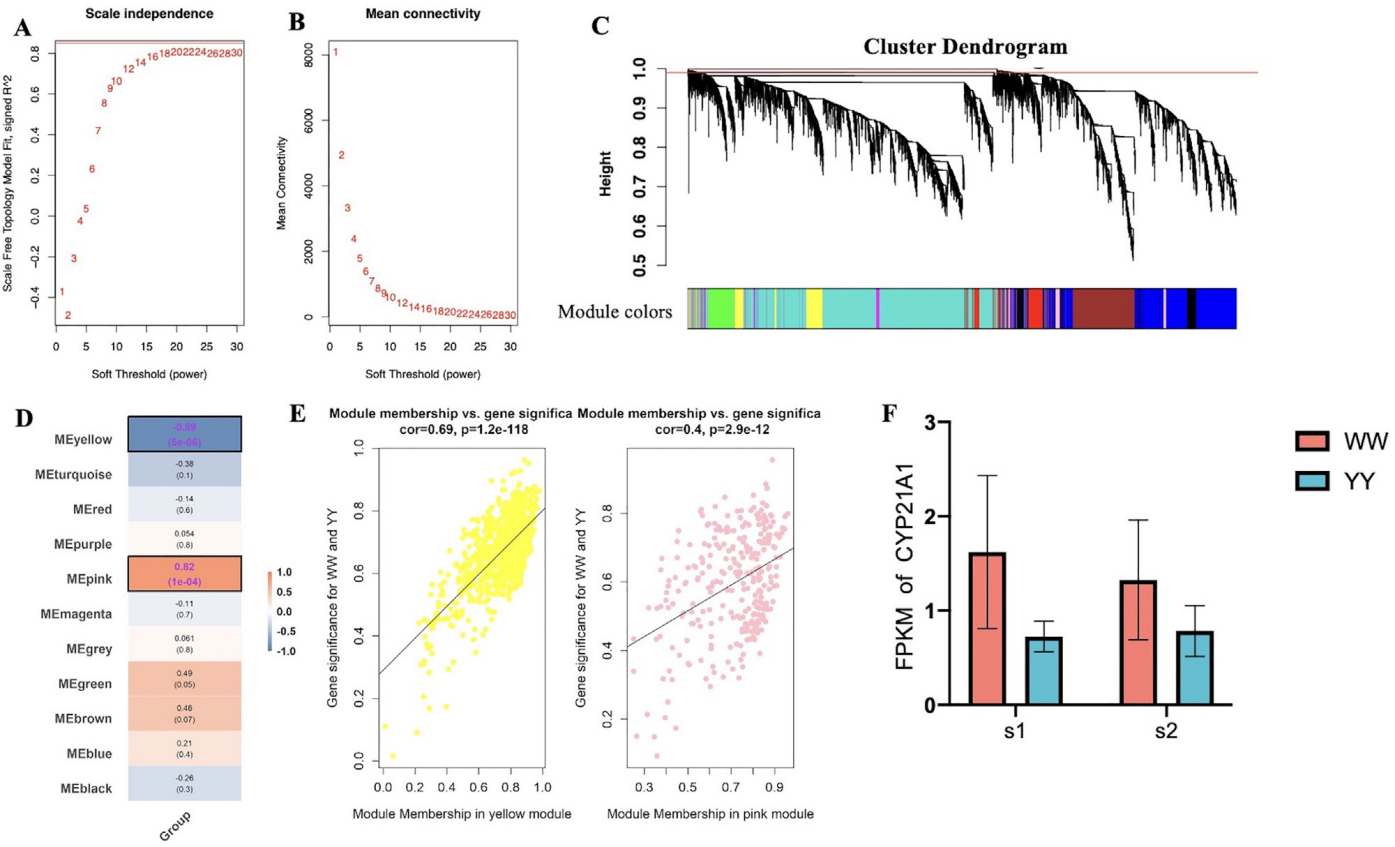
WGCNA for ovarian transcriptome. Analysis of the scale-free fit index for various soft-thresholding powers (A) and analysis of the mean connectivity (B) for various soft-thresholding powers. (C) Co-expression clusters with corresponding color assignments. (D) Module–trait relationship. Each row represents a module eigengene and. Each cell includes the corresponding correlation and *P*-value in the bracket. (E) Scatter plot for gene significance vs. module membership in the yellow and pink module. (F) The expression level of candidate gene CYP21A1 of 2 breeds in 2 laying periods, WW and YY represent White Leghorn and Beijing You chicken, respectively; s1 and s2 denote sexual maturity and peak laying periods, respectively.

WGCNA was used to analyze the expression of 15,627 genes (top 75% MAD genes) for all birds. Based on a scale-free R^2^, the soft-thresholding power was set as 16 (Figure 2A and 2B). Eleven modules were identified when the “mergeCutHeight” was set as 0.25 to merge dynamic modules (Figure 2C). The correlation analysis between the eigengenes of modules and groups showed that the pink module exhibited the highly positive correlation (r = 0.82, *P* < 0.01) with egg production, whereas the yellow module exhibited the highest negative correlation with egg production (r = -0.89, *P* < 0.01) (Figure 2D). We further focused on the genes in pink and yellow modules for scattering plots of GS versus MM (the correlation between the expression profile of a sample and the ME expression profile of associated gene of a certain feature). In the 2 modules, we found a significant correlation between GS and MM, indicating that genes in the modules were highly correlated with egg production (Figure 2E). To obtain a deeper insight into the functions of these associated genes, GO and KEGG pathway enrichment analysis were performed for the annotated genes (225 genes in pink and 467 genes in yellow) in the 2 modules. For the pink module, 3 significant GO terms including 2 CCs and 1 BP were identified. For the yellow module, twelve significant GO terms including 3 BPs, 7 CCs and 2 MFs were enriched (Supplementary Table S4). Regarding KEGG pathway, valine, leucine and isoleucine degradation pathway and metabolic pathway were significantly enriched by pink and yellow module genes, respectively (Supplementary Table S5). In addition, few GO terms and pathways enriched by module genes suggested that egg production traits were complex regulated by multiple genes. *CYP21A1* that harbored in the pink module was involved in the steroid hormone biosynthesis pathway, which was not significantly enriched. We further analyzed the genes with the highest connectivity (hub genes) in the modules (Supplementary Table S6) and found that *CYP21A1* was a hub gene (MM=0.59, GS= -0.64). Moreover, the expression level of the *CYP21A1* in White Leghorns was higher than that of Beijing You chicken during the 2 laying periods (Figure 2F).

### Effect of FSH on CYP21A1 Expression

**Figure 3:**
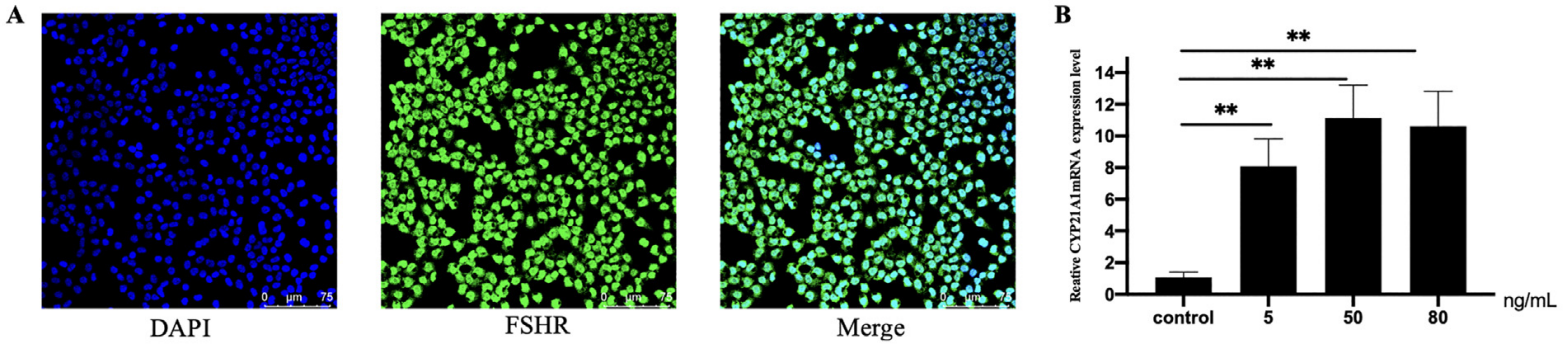
Identification of granulosa cells and the FSH treatment. (A) Immunofluorescence analysis was performed to identify chicken granulosa cells. FSHR: green, granulosa cells marker; DAPI: blue, nucleus; Merge: identification of granulosa cells. (B) Expression of *CYP21A1* mRNA is affected by FSH treatment in granulosa cells of chicken pre-hierarchal and hierarchal follicles. Data are presented as mean ± SEM. *P < 0.05, **P < 0.01.

We collected chicken granulosa cells from the granular layer of the hierarchical follicles. By conducting the immunofluorescence assay, we demonstrated that the cell type was granulosa cells, in which FSHR was expressed and localized in the cytoplasm of granulosa cells (Figure 3A). Then, the granulosa cells were treated with different concentrations of FSH (5, 50, 80 ng/mL) to detected the gene expression of *CYP21A1*. The results showed that FSH could stimulate the expression of *CYP21A1*, and different concentrations of FSH significantly (*P* < 0.01) increased the expression level of *CYP21A1* (Figure 3B), suggesting that *CYP21A1* was regulated by FSH hormone in granulosa cells.

### CYP21A1 Promotes Proliferation and Inhibits Apoptosis in Ovarian Granulosa Cells

**Figure 4:**
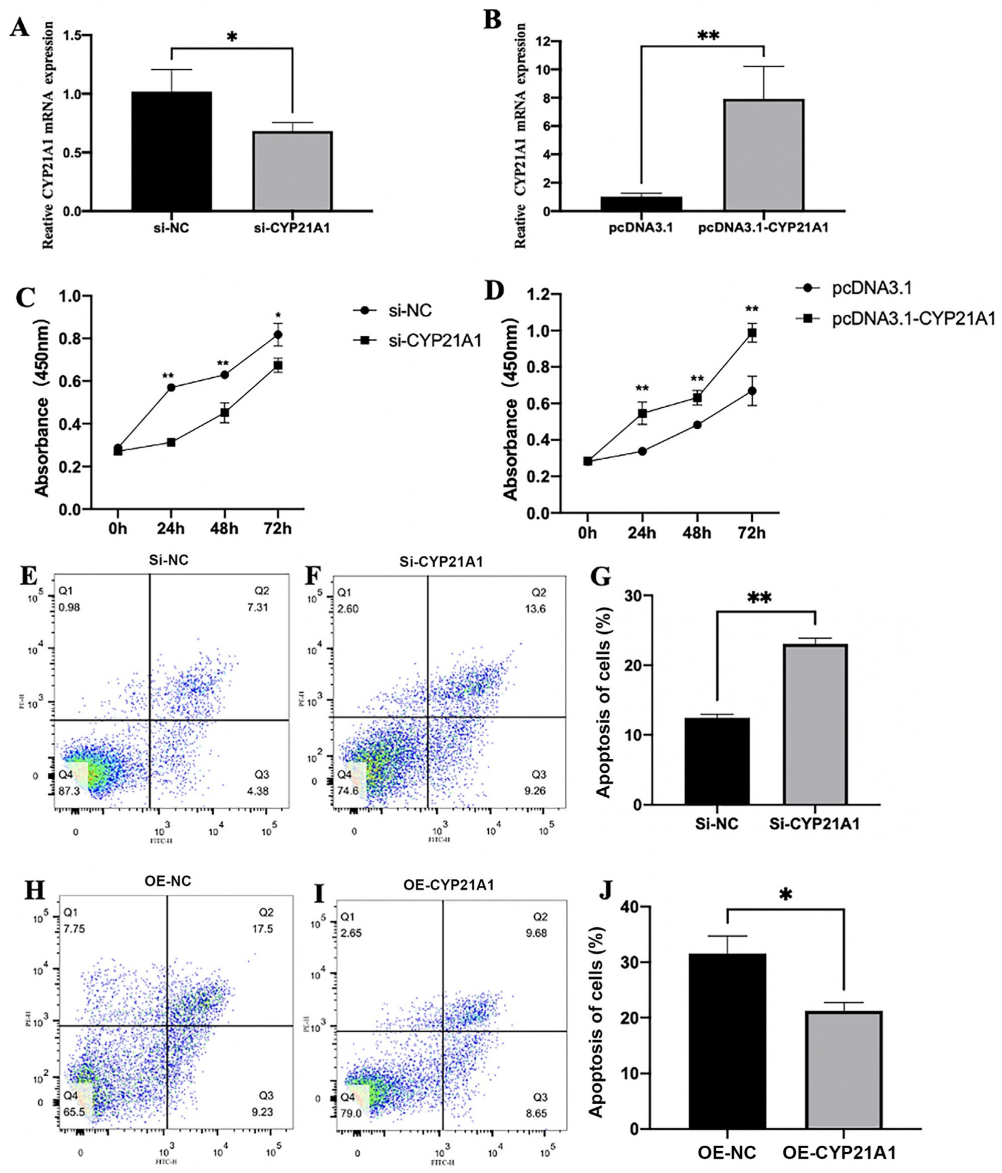
*CYP21A1* promotes the proliferation of chicken granulosa cells and inhibits the apoptosis of chicken granulosa cells. The qRT-PCR results of the *CYP21A1* after the overexpression or interference of *CYP21A1*(A, B). The chicken granulosa cells proliferation curves were measured with CCK-8 assay after the overexpression or repression of *CYP21A1*(C, D). Flow cytometry results of annexin V-FITC/PI staining of the chicken granulosa cells after *CYP21A1* overexpression or interference (E, F, H, I). The apoptosis rate of chicken granulosa cells after the overexpression or repression of *CYP21A1* (G, J). Data are presented as mean ± SEM. *P < 0.05, **P < 0.01.

To study the function of *CYP21A1* in the chicken ovarian follicular development, we constructed a *CYP21A1* overexpression vector and synthesized *CYP21A1*-specific siRNA. The pcDNA3.1-EGFP of *CYP21A1* plasmid and *CYP21A1*-specific siRNA were transfected into the granulosa cells. The results of qRT–PCR indicated that *CYP21A1*-siRNA significantly silenced the expression of *CYP21A1* in chicken granulosa cells (*P* < 0.05, Figure 4A). Conversely, the expression of *CYP21A1* was significantly elevated after transfecting with pcDNA3.1-EGFP-*CYP21A1* (*P* < 0.01, Figure 4B). We later tested the effects of *CYP21A1* on the proliferation of granulosa cells using the CCK-8 kit. We found that interference of *CYP21A1* by specific siRNA significantly decreased granulosa cells proliferation at 24, 48 and 72h (*P* < 0.05, Figure 4C). The overexpression of *CYP21A1* by the recombinant plasmid vector pcDNA3.1-EGFP-*CYP21A1* significantly stimulated the proliferation of granulosa cells at 24, 48 and 72h (*P* < 0.01, Figure 4D). Furthermore, we investigated the effects of *CYP21A1* on granulosa cells collected at 48h after transfection by measuring cell apoptosis with flow cytometry. The number of apoptotic cells in the siRNA-*CYP21A1* group was 23.07% (Figure 4F), which was significantly higher than that in the negative control (**NC**) (12.46%; P < 0.01; Figure 4E and 4G). The number of apoptotic cells in the pcDNA3.1-*CYP21A1* group was 21.25% (Figure 4I), which was significantly lower than that in the NC group (31.54%; *P* < 0.05; Figure 4H and 4J).

### CYP21A1 Regulates Steroid Hormone Production in Chicken Granulosa Cells

**Figure 5:**
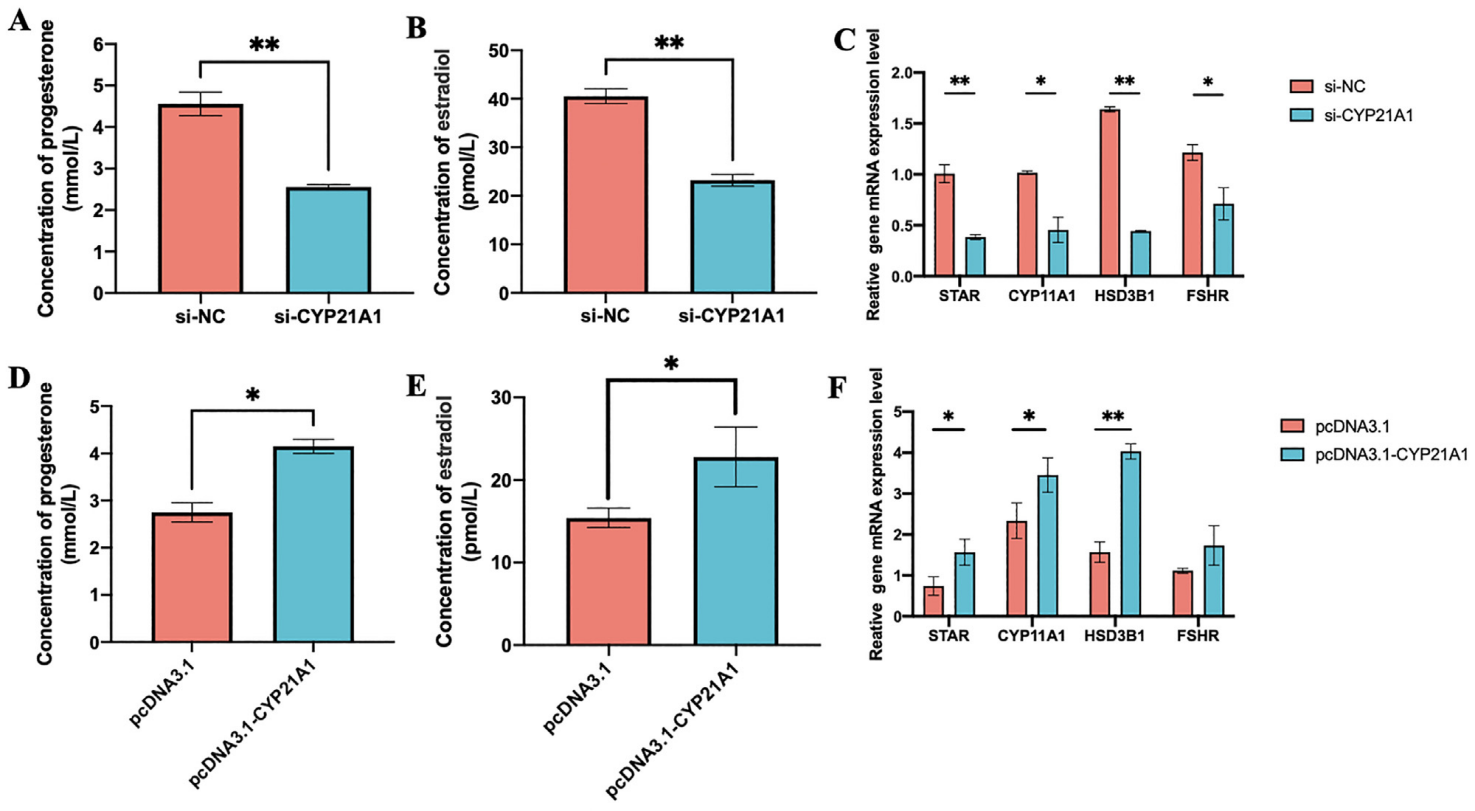
*CYP21A1* promotes the synthesis of steroid hormones in chicken granulosa cells. Effects of interference and overexpression *CYP21A1* on progesterone secretion by chicken ovarian granulosa cells (A, D). Effects of interference and overexpression *CYP21A1* on estradiol secretion by chicken ovarian granulosa cells (B, E). Effects of interference and overexpression *CYP21A1* on the mRNA expression of several key genes involved in steroid hormone synthesis (C, F). Data are presented as mean ± SEM. *P < 0.05, **P < 0.01.

The above results indicated that *CYP21A1* played a regulatory role in the proliferation and apoptosis of granulosa cells. We further tested the function of granulosa cells in regulating the production and secretion of steroid hormones. ELISA results showed that the siRNA of *CYP21A1* significantly reduced the secretion of progesterone and estradiol production (*P* < 0.01, Figure 5A and 5B). The interference of *CYP21A1* repressed the expression of genes related to the follicular development and the synthesis of steroid hormones, such as *STAR, CYP11A1, HSD3B1* and *FSHR* (*P* < 0.01, Figure 5C). Conversely, the overexpression of *CYP21A1* promotes expression of *STAR, CYP11A1, HSD3B1* and the production and secretion of steroid hormones (*P* < 0.05, Figure 5D–5F).

## DISCUSSION

The persistent development of ovarian follicles and ovulation determine the laying efficiency of hens. Egg laying process is mainly regulated by the hypothalamic-pituitary-ovarian axis involving a series of sexual steroid hormones, genes, and interactions among them, which requires delicate regulations at both the transcriptional and post-transcriptional levels (Etches et al., 1983; Bronneberg et al., 2009; Onagbesan et al., 2009; Brady et al., 2019). As a result, exploring gene expression patterns during the development of the follicle is crucial to understanding the regulatory mechanism of laying performance. As an important component of the ovarian follicles, the growth of granulosa cells is closely related to the structural integrity and developmental process of the follicles. Many studies reported high-throughput transcriptomic sequencing is efficient method in identifying differentially expressed genes in follicular development (Onagbesan et al., 2009). However, the functional mechanism of candidate genes is rarely reported.

In the present study, we identified 410 commonly upregulated genes in 2 laying periods using the RNA sequencing method. Functional enrichment analysis showed that commonly upregulated genes are mainly enriched in GO terms, such as endodermal cell differentiation, multicellular organism development, and cell adhesion. This suggests that commonly upregulated genes are closely linked to the growth and differentiation of chicken ovarian follicles. These were consistent with previous studies (Sun et al., 2022; Zhu et al., 2023). The KEGG enrichment analysis revealed 3 common pathways that significantly enriched, such as the ECM-receptor interaction pathway and focal adhesion. The wall of the hen's follicles is mainly composed of extracellular matrix (**ECM**), which comprises collagenous fibers, dermatan sulfate, heparan sulfate, elastin, and hyaluronic acid, governing the proliferation, differentiation, and function of cells (Smith et al., 2002; Hrabia, 2021). Previous study suggested that the ECM-receptor interaction pathway is essential for follicular development in chicken (Sun et al., 2022). Regarding the focal adhesion, previous analyses in zebrafish (Chen et al., 2019), pig (Xu et al., 2015), goat (Su et al., 2018), duck (Tao et al., 2017) and chicken (Zhang et al., 2019a) have demonstrated the important role of focal adhesion pathway in controlling ovarian function, egg-laying performance and other reproductive activities. Moreover, several studies have shown that steroid hormone biosynthesis played a regulatory role in follicular development in pig (Mlyczyńska et al., 2023) and sheep (Venkata Ratna et al., 2023). Herein, *CYP21A1* was enriched in this pathway, which was presumed to be related to follicle development.

How to screen the key genes and their interaction mechanism is always a challenge in the traditional single-dimensional study of quantitative traits. WGCNA may be an effective method to identify valuable expression data for analyzing the intricate genetic network (Langfelder and Horvath, 2008). It transforms the associations between thousands of genes and phenotypes into associations between multiple gene sets and phenotypes, which can not only effectively reflect the interactions between genes, but also do not require multiple hypothesis testing and corrections (Xing et al., 2021). Herein, gene expression network was constructed using a scale-free network to strengthen strong correlations and weaken weak correlations. The proper soft threshold (**SFT**) is selected to make the constructed network resemble to the scale-free network. Generally, the higher the R^2^ value, the better the biological significance. The SFT was set when the R^2^ reached the maximum value 0.8. The mean connectivity also indicated a high correlation between genes in the module and thus laying a foundation for locating key genes. Subsequently, we identified 2 modules that were significantly correlated with egg production. Functional enrichment analysis showed significant differences in gene function between the 2 significant modules, which were all involved in ovarian function and egg production. The genes in the pink module were significantly enriched in the valine, leucine and isoleucine degradation pathway which were closely associated with energy metabolism (Xu et al., 2020). *CYP21A1* was identified as a hub gene in the pink module, further indicated the role of gene played in the ovary affecting the egg production. In addition, few GO terms and pathways enriched by hub genes suggested that egg production traits were complex governed by multiple factors.

Follicle-stimulating hormone receptor is specifically expressed in the follicular granulosa cells of animals. Immunofluorescence identification by *FSHR* antibodies revealed positive expression of cytosolic *FSHR* in follicular granulosa cells, confirmed that granulosa cells are the representative cell types that expressed *FSHR* (Verbraak et al., 2011; Du et al., 2016). Reproductive hormones are known to play an important role in the regulation of follicle development (Peretz et al., 2011), which is strictly dominated by FSH through binding to its receptor *FSHR* (You et al., 1996). In this study, FSH stimulation at different concentrations could significantly increase the expression level of *CYP21A1* (*P* < 0.01), suggested the potential role of *CYP21A1* as a functional gene regulating follicular development. This was also reported for functional genes in human and chicken granulosa cells (Baumgarten et al., 2015; Wang et al., 2017; Li et al., 2019; Zhong et al., 2021). Consequently, we investigated the role of *CYP21A1* in granulosa cells.

The proliferation and apoptosis of granulosa cells are closely related to ovarian follicular development (Vanderhyden et al., 1992; Macklon and Fauser, 1999). Herein, we found that overexpression of *CYP21A1* significantly promoted the proliferation and inhibited the apoptosis of GCs by cell proliferation and apoptosis assays. By contrast, when *CYP21A1* was interfered, the proliferative capacity of GCs was significantly decreased and the apoptosis rate was significantly increased. Thus, it demonstrated that *CYP21A1* had a promotional effect on follicle development in chicken. Chicken reproduction is heavily regulated by sex steroid hormones (Johnson et al., 2002), such as progesterone and estradiol, which are involved in the regulation of ovulation, gonadal differentiation, and sexual and nesting behaviors in birds through interactions with their intracellular receptors (Ghanem and Johnson, 2019; Liu et al., 2019). Steroid hormones are synthesized from cholesterol through a series of enzymatic reactions, in which *STAR, CYP11A1* and *HSD3B1* were essential to this conversion (Lee et al., 1998). In terms of biochemistry function, *CYP21A1* converts progesterone and 17α-hydroxyprogesterone into deoxycorticosterone and deoxycortisol, respectively, within metabolic pathways that ultimately lead to aldosterone and cortisol, which are essential to produce steroid in maintaining the ovary function (Heikkilä et al., 2002; Bidet et al., 2010; Amin et al., 2014). Herein, overexpression of *CYP21A1* in chicken granulosa cells increased the expression of *STAR, CYP11A1* and *HSD3B1*. Conversely, the knockdown of *CYP21A1* in chicken granulosa cells inhibited the expression of *STAR, CYP11A1, HSD3B1* and *FSHR*, suggested that the ovarian follicle expressed more *CYP21A1*, and stimulates the expression of *STAR, CYP11A1* and *HSD3B1* to enhance the conversion from cholesterol to the steroid hormones. As expected, the ELISA experiment also showed increased and decreased progesterone and estradiol accumulation in the *CYP21A1* overexpressing and interfering groups, respectively. Therefore, *CYP21A1* acted as a positive regulator of ovarian follicle growth and development by altering the expression of steroid hormone synthesis-related genes and the synthesis of steroid hormone in the hen ovary. Furthermore, it needs to be further studied for how large the effects of *CYP21A1* played on the cells compared to *CYP11A1* and *HSD3B1*, and the underlying regulatory mechanism that the metabolites of *CYP21A1* on the follicle development and steroid hormone synthesis.

## CONCLUSIONS

In summary, the transcriptome analysis revealed that *CYP21A1* was an up-regulated hub gene in White Leghorns compared to Beijing You chicken during sexual maturity and peak laying periods. Combined with the molecular experiment, we demonstrated that *CYP21A1* regulated ovarian follicle development by promoting the proliferation of granulosa cells, inhibiting the granulosa cells apoptosis, and increasing the secretion of progesterone and estradiol. These findings would contribute to the understanding of a novel function of *CYP21A1* in the ovarian follicle development of chickens.

## Appendix Supplementary materials

Image, application 1

## Footnotes

Supplementary material associated with this article can be found, in the online version, at doi:10.1016/j.psj.2024.103589.
